# Signal-specific performance of in-ear EEG: strengths and limitations

**DOI:** 10.3389/fnins.2026.1859327

**Published:** 2026-07-29

**Authors:** Vanessa Frei, Pablo Mainar, Thomas Fritz, Jean-Michel Chardon, Nathalie Giroud

**Affiliations:** 1 Department of Psychology, University of Florida, Gainesville, FL, United States; 2 McKnight Brain Institute, University of Florida, Gainesville, FL, United States; 3 Logitech S.A., Lausanne, Switzerland; 4 Department of Informatics, University of Zurich, Zurich, Switzerland; 5 Neurosciences of Communication, Language, and Cognition, University Hospital of Psychiatry Zurich, Zurich, Switzerland; 6 International Max Planck Research School on the Life Course: Evolutionary and Ontogenetic Dynamics (LIFE), Dynamics, Berlin, Germany; 7 Language and Medicine Center Zurich, Competence Center of Medical Faculty and Faculty of Arts and Sciences, University of Zurich, Zurich, Switzerland; 8 Neuroscience Center Zurich, University of Zurich and ETH Zurich, Zurich, Switzerland

**Keywords:** auditory ERP, EEG, EEG validation, in-ear EEG, speech-in-noise

## Abstract

Fully in-ear Electroencephalography (EEG) configurations prioritize wearability and rapid setup but may constrain spatial sampling and signal-to-noise characteristics. We conducted a domain-specific evaluation of a generic-fit, fully intra-auricular dry-electrode EEG system benchmarked against simultaneous 32-channel BioSemi scalp EEG in 19 healthy adults. Rather than testing equivalence, we mapped strengths and boundary conditions across (i) resting-state alpha modulation (eyes open vs. eyes closed), (ii) auditory event-related-potentials (ERP; N1-P2 complex), and (iii) alpha ERSP during speech-in-noise. The in-ear configuration robustly captured canonical resting-state alpha increases during eyes-closed periods, despite reduced absolute amplitude relative to scalp EEG. In contrast, reliable N1-P2 responses were observed only in conventionally average-referenced scalp EEG; in-ear and adapted referencing configurations showed reduced component detectability with lower component SNR. During speech-in-noise, significant alpha ERSP deviations from baseline emerged in scalp EEG under less adverse listening conditions, whereas comparable deviations were not consistently detected in the minimal in-ear configuration. These findings indicate that signal detectability depends strongly on signal class, effect size, and recording geometry, providing configuration-aware guidance for study design and future in-ear EEG development.

## Introduction

Electroencephalography (EEG), a technique capturing electrical brain activity by electrodes placed on the scalp, has been well established over the past century and proven to be an indispensable method for both basic and applied neuroscience as well as clinical practice (e.g., in epilepsy) (Teplan, 2002). The traditional EEG consists of multiple electrodes systematically distributed and secured around the scalp, while an electrolyte gel is applied to increase conductivity between skin and electrode. This approach enables capturing high-quality signals with great temporal resolution in the (sub)millisecond range, allowing to draw conclusions about the temporal occurrence of processing patterns within the brain (Pfurtscheller and Lopes da Silva, 1999). However, to ensure data quality, EEG assessments are often scheduled in highly controlled laboratory environments lacking ecological validity. As such, the need has emerged to develop and evaluate new methods that allow for a robust assessment of neural activity outside of the laboratory context.

The possibility of long-term monitoring in more naturalistic settings holds great potential for understanding neural dynamics in real-world environments (Casson, 2019; Kaongoen et al., 2023; Mikkelsen et al., 2015). Recording outside the laboratory environment can provide valuable insights into mental health (e.g., stress-induced changes, emotion monitoring), cognitive load (e.g., school tasks, workplace effort) as well as individual changes over time by facilitating the design of interventions tailored to individual need (Sidelinger et al., 2023; Hu et al., 2011). Based on the growing interest in exploring neural activity in more naturalistic environments, paired with technological advancements, a research field has developed, with a specific focus on reduced EEG systems. As such, the ear has become an area of interest in this research field, as it provides an ideal location for measuring neural activity with a reduced EEG system. Ear EEG, initially introduced by Looney et al. (2011), represents a new acquisition technique by placing electrodes in or around the ears capturing neural activity (Kidmose et al., 2012; Debener et al., 2015). The general idea of ear EEG is to simplify the setup, increasing accessibility and wearability, and therefore promoting monitoring of neural activity in naturalistic or clinical environments (Kaongoen et al., 2023).

Ear EEG systems represent an innovative extension of conventional EEG methodology. They have been successfully designed, implemented, and validated in a range of applications, offering novel opportunities for portable and user-friendly neurophysiological assessments. However, the reduced spatial sampling and modified hardware configuration inherent to such systems introduces specific challenges in signal acquisition and robustness that warrant careful methodological consideration. Regarding the hardware, several designs have been introduced, differing in multiple important aspects: Placement (within vs. around the ear), number of electrodes, fit (individually tailored vs. generic), used materials (silicone vs. viscoelastic earpieces) and types of electrodes (for a comprehensive documentation of the currently used designs of ear EEG system see a review by Kaongoen et al. (2023).

In-ear EEG systems have emerged as a promising extension of mobile neurophysiology, offering discreet and wearable alternative to conventional multi-channel scalp EEG. However, hardware implementations vary substantially across studies, particularly with respect to electrode placement, referencing strategy, and the use of additional external electrodes. Some systems combine in-ear electrodes with supplementary scalp electrodes around the ear (Debener et al., 2015; Bleichner and Debener, 2017; Wang et al., 2012), whereas others rely exclusively on electrodes embedded within the ear for both signal acquisition and referencing (Mikkelsen et al., 2015; Wu et al., 2020; Fiedler et al., 2017). These design differences are not merely technical details, they directly influence spatial sampling, signal-to-noise characteristics, and overall robustness of the recorded data.

Although ear-EEG captures the same underlying volume-conducted cortical activity as conventional scalp EEG, the physiological characteristics of the recorded signal differ due to the altered recording geometry. Intra-auricular electrodes are positioned closer together and sample electrical fields from a more restricted spatial region than conventional scalp arrays. Consequently, recorded amplitudes are typically reduced and sensitivity to specific cortical generators may differ depending on their anatomical location and orientation. Signals originating from sources located near temporal regions may remain relatively accessible, whereas activity generated by more distant cortical regions may be attenuated. These physiological constraints do not preclude the measurement of neural activity but imply the signal detectability may vary substantially across EEG paradigms and recording configurations.

In the present study, we evaluate a fully in-ear EEG configuration (provided by Logitech S.A.) in which all recording, reference, and ground electrodes are located within the ear and no additional external electrodes are used. The device employs a generic (non-individualized) fit and is designed to prioritize wearability, minimal setup, and ease of use without requiring expert electrode placement. Such a reduced configuration represents an important design direction for scalable and user-friendly mobile EEG applications. At the same time, limiting electrode number and spatial coverage may constrain the range of neural signals that can be reliably captured.

Rather than testing the general validity of in-ear EEG as a concept, which has been demonstrated in prior work (Kaongoen et al., 2023), the current study focuses on evaluating the signal characteristics of this specific minimal in-ear implementation. Our goal is to determine which commonly used EEG markers can be robustly detected with a generic, fully in-ear configuration and where potential limitations may arise. By systematically comparing performance across paradigms of varying complexity and directly benchmarking results against a conventional scalp EEG system, we aim to delineate the practical strengths and constraints of this reduced hardware design. These findings are intended to inform future study design, task selection, and system development decisions when employing generic, fully in-ear EEG configurations.

To assess the performance of this device relative to a conventional scalp EEG system, we selected paradigms that vary in their physiological and analytical demands. Resting-state alpha modulation during eyes-open versus eyes-closed periods represents a robust and well-established spectral marker that is frequently used to monitor neural state changes and evaluate signal quality. Auditory event-related potentials (ERPs), specifically the N1–P2 complex elicited by pure tones, provide a benchmark for assessing the temporal precision and signal-to-noise characteristics of time-locked responses. Finally, alpha power modulation during a speech-in-noise listening task was included to examine whether the in-ear device can capture frequency-band changes associated with more complex and ecologically valid cognitive demands. For each paradigm, recordings obtained with the in-ear system were directly compared to data acquired using a 32-channel Biosemi scalp EEG system. Because several target signals, particularly alpha oscillations, exhibit pronounced spatial topographies, scalp EEG data were evaluated using both a conventional average-reference approach and an adapted temporal configuration (T8 referenced to T7) designed to more closely approximate the spatial sampling geometry of the in-ear device. This dual-comparison approach allowed us to distinguish between differences attributable to the recording modality itself and those arising from spatial sampling constraints. Using these paradigms, we addressed the following research questions:

- Can a fully in-ear, generic-fit EEG device reliably capture basic spectral state changes such as alpha attenuation during eyes-open versus eyes-closed resting state conditions?
- To what extent does the temporal precision of auditory ERPs recorded with the in-ear device resemble those obtained with a conventional 32-channel scalp EEG system?
- Does the performance of the in-ear device differ between basic spectral measures (resting-state alpha), time-locked responses (auditory ERPs), and more complex task-related modulations (listening effort)?

Rather than assuming equivalence with traditional scalp EEG, our aim was to delineate the strengths and boundary conditions of this reduced hardware configuration. In doing so, we aimed to move beyond a binary question of feasibility and instead characterize the practical trade-offs inherent to a minimal, fully in-ear design. By identifying where such a configuration performs robustly and where performance may be constrained, we provide empirically grounded guidance for study design, task selection, and future system development in mobile EEG research.

## Materials and methods

### EEG systems

The present study was designed as a functional performance comparison in a controlled laboratory environment rather than as a dedicated electrical hardware characterization. Specifically, we evaluated a minimal, generic-fit, fully in-ear EEG configuration in its intended dry-electrode setup and benchmarked its performance against a conventional multi-channel scalp EEG system across paradigms of varying signal demands.

Scalp EEG (reference system): Continuous EEG was recorded using a 32-channel BioSemi ActiveTwo system with Ag/AgCl electrodes (BioSemi, Amsterdam, The Netherlands) at a sampling rate of 2,048 Hz, while an online filter between 0.1 and 100 Hz was applied.

**FIGURE 1:**
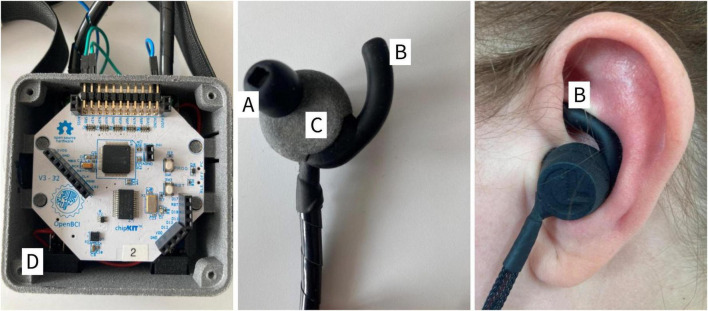
In-ear EEG device and signal acquisition board. **(A)** Active signal electrode (right) or reference electrode (left). **(B)** Passive grounding electrode placed within the concha. **(C)** 3D printed body. **(D)** 8-channel Cyton Biosensing Board.

In-ear EEG (test system): The in-ear system (custom-built by Logitech S.A.) employed a fully in-ear electrode configuration without external electrodes. Two active dry electrodes were used for signal acquisition and referencing (signal electrode in the right ear canal; reference electrode in the left ear canal; containing a small amplifier in the body of the earbud to ensure signal transmission to signal processing module), and a passive ground electrode was placed in the right concha. Electrodes were encased in conductive polymer and embedded in a 3D-printed generic (non-individualized) earbud body (Figure 1). Neural signals were digitized using an OpenBCI Cyton biosensing board (8-channel) incorporating a Texas Instruments ADS1299 biopotential analog front-end (24-bit delta-sigma ADC with integrated programmable gain amplifier). Data were streamed wirelessly to the recording computer. Consistent with Cyton streaming constraints, data were acquired at 250 Hz sampling rate. Online visualization was performed using the OpenBCI GUI (OpenBCI GUI, version 4.1.5); note that GUI filters are applied for real-time display only, whereas recorded data are stored as raw, unfiltered signals for offline processing. Experimental triggers were synchronized via a dedicated physical trigger line rather than Bluetooth transmission to preserve temporal alignment between stimulus events and recorded data.

Electrode contact quality was assessed prior to each recording by verifying stable signal traces and minimizing obvious movement artifacts. Electrode–skin impedance was not quantified at the start or end of recordings. Although impedance/lead-off measurement can be implemented on ADS1299-based systems, this was not part of the current acquisition protocol. As a consequence, the present results reflect performance under typical dry-contact use rather than a best-case, gel-optimized configuration. Accordingly, conclusions from this work pertain to the functional performance and boundary conditions of this specific minimal, generic-fit, fully in-ear dry-electrode implementation when benchmarked against a standard 32-channel scalp EEG system. The study was not designed to dissociate limitations inherent to in-ear EEG technology in general from those specific to this hardware configuration. Data preprocessing was performed using Python (version 3.12.4) and the MNE-Python package (Gramfort et al., 2013).

### Sample

To compare the in-ear device to the Biosemi system across varying standardized EEG paradigms, a sample of 19 healthy young individuals were tested (M_*Age*_ = 32, Range_*Age*_ = 19–51, *SD*_*Age*_ = 7.94, female = 13). Participants did not report any pre-existing neurological or psychological conditions or any speech or language pathology. All participants completed the full protocol. However, data inclusion varied by task and recording configuration due to preprocessing-based quality control and availability of complete task segments. While all 19 participants contributed usable BioSemi resting-state data, usable sample sizes ranged from 16 to 19 across tasks and configurations. Final sample sizes are reported separately for each analysis in the corresponding results section. All participants were Swiss German or German native speakers and did not report any hearing impairment. Participants were paid CHF 20 per hour, with the average time spent on the experiment being 1.5 h. Informed consent was obtained from all participants. The study was conducted ethically, in compliance with the Declaration of Helsinki and approved by the Ethics Committee of the Faculty of Arts and Social Sciences of the University of Zurich (approval number 24.08.05).

### Procedure

For the current project, neural activity was measured while being exposed to three varying tasks, namely a resting-state, auditory stimulation and speech in noise task. Experimental stimuli were programmed and presented using PsychoPy (Peirce, 2007).

#### Resting-state task

The experimental procedure started with resting-state EEG recording under two conditions: eyes closed and eyes open. Participants were seated in an electrically shielded sound treated booth in front of a screen. Participants were informed to close their eyes and relax, while neural resting-state activity was recorded over a period of 3 min. An auditory stimulation delivered through loudspeakers indicated the end of the first task and informed the participants to open their eyes. The second condition captured neural resting-state activity while the eyes were kept open. Participants were instructed to focus on a white fixation cross on a black background for the next 3 min. The complete task duration was approximately 8 min. Monitoring varying eye states provides an ideal task design to evaluate the robustness of an in-ear EEG device, given the well-established response observable from a traditional scalp-EEG. When eyes are kept open, a significant alpha attenuation response within the neural signal should be observable, whereby alpha band power (8–13 Hz) decreases compared to eyes closed (Barry et al., 2007; Klimesch, 1999). Furthermore, this dynamic within alpha band power as a function of eye state has also been reported among studies using in-ear EEG systems (Paul et al., 2022a; Paul et al., 2022b; Kaveh et al., 2020; Sintotskiy and Hinrichs, 2020), thereby presenting an ideal task to compare both types of EEG systems.

#### Auditory evoked potential

Auditory event-related potentials (ERPs) involve specific changes in the neural signal in response to an auditory stimulus, with this response being classified into different components based on their polarity and latency. While there are varying components associated with an auditory ERP, the most robust and sensory driven components are the N100, a negative peak at approximately 100 ms after stimulus presentation, and a P200, a positive peak at approximately 200 ms after stimulus presentation. This N1-P2 complex has been well-established within EEG research (Näätänen and Picton, 1987) and characterized by high temporal precision. Therefore, auditory ERPs are deemed a useful task to evaluate the temporal accuracy of an in-ear EEG system (Meiser and Bleichner, 2022; Denk et al., 2018; Valentin et al., 2021; Kaongoen and Jo, 2017). To derive auditory ERPs, participants were presented with 200 repetitions of a sinusoidal tone at 1 kHz. The tone was presented for 100 milliseconds at a sound pressure level of 70 dB trough a centrally placed KEF Q150 loudspeaker (KEF, Maidstone, England). To minimize habituation effects, a stimulus onset asynchrony of 1,000–1,300 ms (300 ms jitter) and an interstimulus interval of 900–1,300 ms was chosen. The complete task duration was approximately 6 min.

#### Speech in noise

To evaluate the robustness of the in-ear EEG device in a more naturalistic and ecologically valid setting, a speech in noise task was presented. Participants listened to natural, continuous speech that was presented with varying intensity of background noise and answered a comprehension question after each sentence. A total of 45 sentences, spoken in standard German, were presented at three different noise levels: no noise, low noise (SNR: 8 dB) and high noise (SNR: 3 dB). The background noise snippets were extracted from stereoscopically recorded cafeteria noise from the HRIR database (Kayser et al., 2009). The sentences were presented at a sound pressure level of 70 dB through a centrally placed loudspeaker. Noise levels changed after every fifth sentence, resulting in 15 sentences per noise level. Comprehension questions, designed to have only one correct verbal answer, were presented on screen after each sentence. A practice session was conducted prior to the experiment to familiarize participants with the task. A speech in noise task was chosen because such a paradigm introduces perceptual and cognitive load that reflects challenges experienced in daily life. The goal was to evaluate if an in-ear EEG system was sensitive enough to capture alpha band modulation within a parietal region, despite the reduced spatial resolution of the device. While this task allows to systematically compare the sensitivity of the in-ear device with a traditional scalp EEG, it further represents a more ecologically valid paradigm, simulating a real-world challenge. This is an important direction, given that the long-term goal of mobile EEG systems is the utilization outside of artificial laboratory settings and capture neural activity in more naturalistic environments.

### EEG data: preprocessing

**FIGURE 2:**
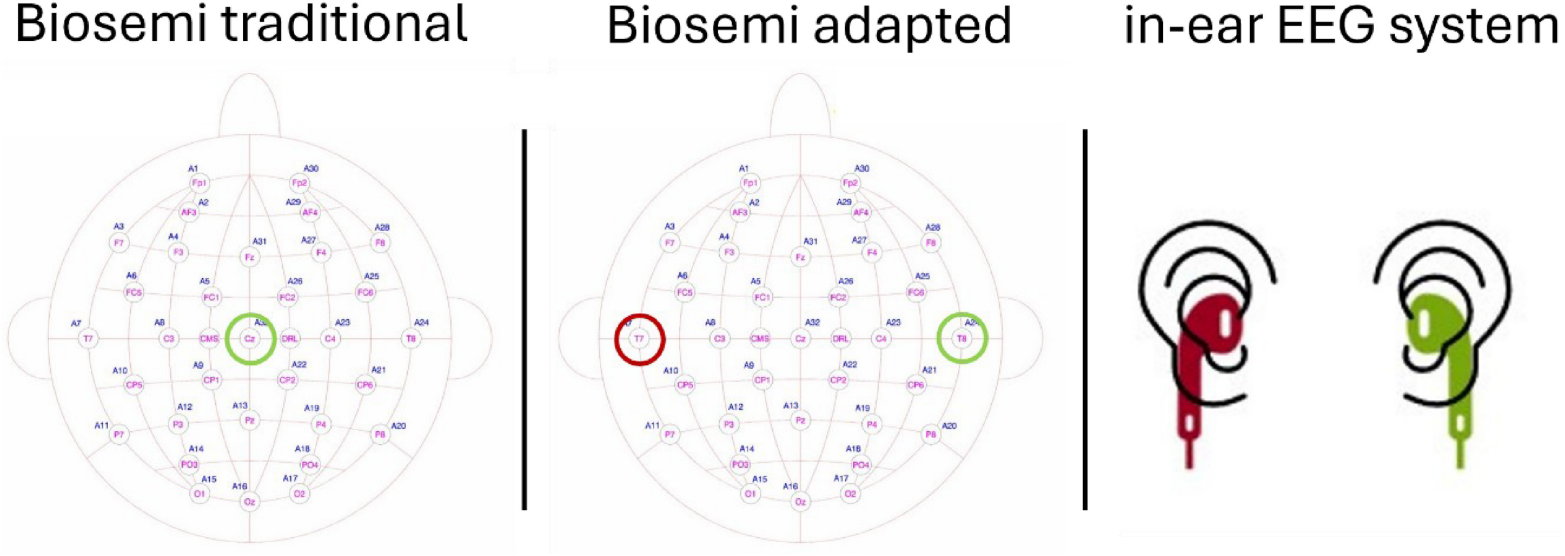
Schematic representation of the varying reference schemes. Depicted are the referencing schemes used for both devices, with red marks indicating the reference electrode, and green marks representing the active electrode.

EEG preprocessing was conducted using standardized procedures appropriate for each recording system, with the goal of ensuring comparable data quality while preserving signal characteristics relevant to the paradigms under investigation. Raw data were examined to identify recordings with exceptionally poor data quality, resulting in a total of three recordings from the in-ear device that had to be rejected (i.e., highly increased noise because of movement). For the Biosemi recordings, 2 different re-referencing schemes were used: Once re-referenced to average (in the following referred to as Biosemi traditional) and once to T7 (reflecting the closest scalp electrode to the in-ear device reference electrode in the left ear). The chosen referencing schemes are schematically visualized in Figure 2. For all the recordings, a bandpass filter between 0.1 and 30 Hz with a third-order Butterworth zero-phase filter to avoid phase distortions and a bandstop-filter was applied between 49 and 51 Hz to control for potential artifacts resulting from electrical interference using a zero-phase IIR filter. For the Biosemi recordings, independent component analysis (ICA) was applied (Jung et al., 2000) to identify and remove ocular artifacts. Equivalent ICA-based correction was not feasible for the in-ear recordings because the device provided only a single EEG channel. Consequently, data quality in the in-ear system was controlled through visual inspection and automated artifact rejection procedures implemented during task-specific processing.

### EEG data: task specific processing

#### Resting-state analysis

Resting-state was recorded for eyes closed and eyes open to quantify the power spectral density within each condition and to use power as a parameter to compare the robustness of the in-ear device to the Biosemi system. Each condition lasted 180 s, with onset times used to segment the EEG data into two epochs: eyes closed and eyes open. Power spectral density (PSD) was computed for each condition using Welch’s method with a 2-s window length (n_fft = 4,096 for the Biosemi system, n_fft = 500 for the in-ear EEG system). The frequency range for the PSD was defined between 0.5 and 30 Hz. For the Biosemi system PSD was once computed for all EEG channels and averaged (Biosemi traditional) to derive a global PSD for each participant, condition, and device. Additionally, PSD was calculated for T8 with data re-referenced to T7 (Biosemi Adapted). For the in-ear EEG device only the active electrode was used. All PSD values were converted to decibels (dB). As a next step, specific frequency bands were defined: Delta (0.5–4 Hz), Theta (4 Hz–8Hz), Lower Alpha (8 Hz–10 Hz), Upper Alpha (10 Hz–13 Hz) and Beta (13 Hz–30 Hz). Frequency band-specific PSD values for each participant, condition and device were used to conduct statistical comparisons. No explicit correction for cardiac artifacts was performed. Given that the primary outcome measure was alpha-band power (8–13 Hz), contamination from cardiac activity was expected to be limited.

#### Auditory evoked potential

Auditory ERPs were quantified to assess whether the minimal in-ear EEG configuration can capture time-locked neural responses to brief acoustic stimulation and how these responses compare to a conventional 32-channel scalp EEG system (BioSemi). From the preprocessed data, 200 event indices (e.g., triggers) were extracted, representing the stimulus onset during the experiment. These triggers marked the beginning of the stimulus presentation and were used to quantify auditory event-related potentials (ERP) from each device.

For both devices, data were band-pass filtered between 0.1 and 30 Hz and notch-filtered at 50 Hz to reduce line noise. Continuous data were segmented into epochs from –200 to 500 ms relative to stimulus onset. Baseline correction was applied using the pre-stimulus interval (–200 to 0 ms). Linear detrending was applied within each epoch. For the BioSemi recordings, two referencing schemes were analyzed: Traditional average reference, with ERP components extracted from Cz and adapted referencing, in which data were referenced to T7 and ERPs were extracted from T8 to approximate the spatial configuration of the in-ear device. For the in-ear device, the active electrode (right ear canal) was referenced to the contralateral ear canal (left ear).

To address potential methodological limitations and ensure that null findings were not attributable to insufficient preprocessing, an automated, data-driven artifact rejection procedure was implemented for both systems. For each participant and configuration, peak-to-peak amplitude was computed for every epoch in the channel of interest. A rejection threshold was determined using a robust estimator defined as: median peak-to-peak amplitude + 5 times median absolute deviation (MAD, scaled to approximate standard deviation). Epochs exceeding these thresholds were excluded from further analysis. For each participant the following quality metrics were computed: Number of detected events, number of epochs retained after rejection, percentage of retained epochs, median peak-to-peak amplitude (μV) and baseline noise level (standard deviation of the pre-stimulus interval). ERP components were quantified using mean amplitude within predefined time windows. Time windows were defined a priori based on canonical latencies and confirmed against the BioSemi grand average for plausibility; the same windows were applied to all configurations. The N1 component was quantified as the mean amplitude within a 125–175 ms post-stimulus window, and the P2 component within a 195–245 ms post-stimulus window. Time windows were centered on peaks identified in the grand-average BioSemi Cz waveform and applied identically across recording configurations. To account for inter-individual differences in noise levels, a signal-to-noise ratio (SNR) metric was computed for each component:


$$S\,N\,R=\frac{\left|c\,o\,m\,p\,o\,n\,e\,n\,t\,a\,m\,p\,l\,i\,t\,u\,d\,e\right|}{b\,a\,s\,e\,l\,i\,n\,e\,s\,t\,a\,n\,d\,a\,r\,d\,d\,e\,v\,i\,a\,t\,i\,o\,n}$$

This metric allowed comparison of component detectability across devices independent of absolute amplitude differences. The data quality and epoch retention metrics are reported in the results section.

#### Speech in noise

To evaluate whether the in-ear device could detect alpha band modulation relative to baseline during speech-in-noise listening, we conducted an event-related spectral perturbation (ERSP) using Morlet wavelets following the approach described by Ala and colleagues (ERSP). Positive ERSP reflects event-related synchronization, while negative ERSP reflects event-related desynchronization. To replicate the authors approach as close as possible, the EEG data from both devices were epoched relative to the onset of each sentence, using a time window of – 4 to 4 s and baseline correction from -4 to 0 s.

### ERSP computation

**FIGURE 3:**
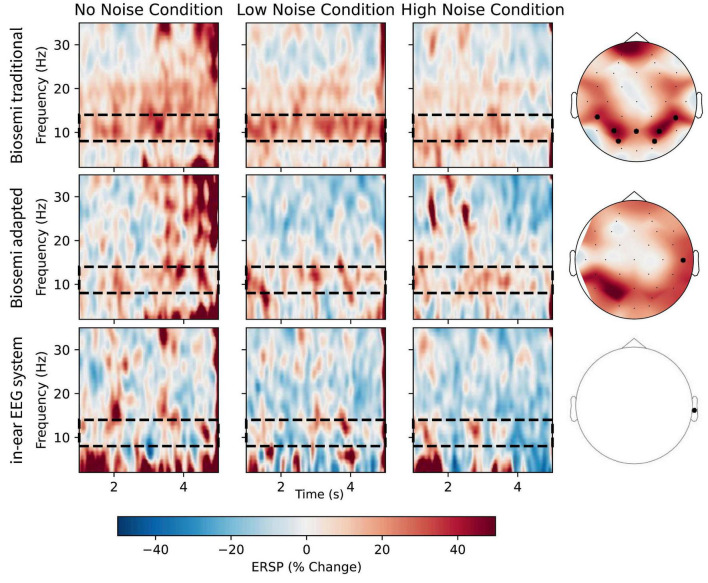
Spectrograms for all three noise conditions and across devices representing ERSP change in percent. Top row depicts the traditional Biosemi system with average re-referencing and across the parietal cluster. Middle row depicts the Biosemi system with adapted re-referencing to T7 and data derived from T8. Bottom row depicts the in-ear EEG system data from the right ear electrode, referenced to the left ear electrode. The dashed line within each spectrogram represents the alpha range from 8 to 14 Hz.

Time-frequency decomposition was performed using Morlet wavelets with a 7-cycle width across 2–35 Hz, centered at 500 ms steps. The ERSP was computed as the relative change in spectral power compared to the pre-stimulus baseline (-4 to 0 s), using the formula:


$$E\,R\,S\,P\,\left(t,f\right)=\frac{A\,\left(t,f\right)-\bar{R\,\left(f\right)}}{\bar{R\,\left(f\right)}}\times 100$$

where *A(t, f)* is the absolute post-stimulus power at time *t* and frequency *f*, and $\bar{R\,\left(f\right)}$ is the absolute power during baseline at the same frequency. The first second of post-stimulus data were excluded to avoid contamination from event-related potentials. Following Ala et al. (2022), alpha power (8–14 Hz) was extracted by averaging spectral power within the parietal electrode cluster (PO4, PO3, Pz, P3, P4, CP5, CP6; visualized in Figure 3) for the scalp EEG with average referencing. For the scalp EEG with adapted referencing, only the data from T8 was used, reflecting a similar spatial resolution as for the in-ear system.

### Statistical analysis

Given the differences in signal power across devices, we decided to not directly statistically compare the data from both devices. Instead, we compared the conditions provided by the task within the device and then assessed if the same results were obtained.

Resting-state EEG data were analyzed to compare PSD between eyes closed and eyes open across five frequency bands. Shapiro-Wilk tests were conducted to assess the normality of the PSD distributions in each condition, resulting in a choice for parametric statistical tests. Since the data exhibited paired observations for each participant across conditions, paired-samples *t*-tests were performed for each frequency band to assess differences in PSD between the two conditions.

ERP data were analyzed to assess the amplitudes of two components of interest: N1 and P2. For each ERP component, one-sample *t*-tests were conducted to determine whether the mean amplitude was significantly different from baseline. This test assessed the presence of reliable ERP responses, with the null hypothesis stating that the mean amplitude did not significantly differ from zero.

Lastly, the analysis of the data collected during the speech in noise presentation was used to evaluate whether alpha power modulation significantly deviated from baseline. One-sample *t*-tests were conducted for each noise condition (no noise, low noise, high noise) separately for each EEG system and referencing scheme. Each participant’s alpha ERSP value was computed by averaging the relative change in spectral power within the 8–14 Hz range, extracted from the parietal electrode cluster (for the Biosemi traditional referencing), from T8 (adapted referencing) or from the right electrode of the in-ear EEG system. A significant result (*p* < 0.05) indicated that the alpha power showed a meaningful deviation (event-related (de)synchronization) from baseline. All statistical analyses were conducted using R version 4.1.2 (R Core Team, 2020). Importantly, no direct statistical comparison between the EEG systems was performed due to the overall difference in signal power. Instead, we evaluated whether alpha modulation was significantly different from the corresponding baseline within each system to assess if the in-ear device could also detect alpha modulation from a parietal region.

## Results

### Resting-state EEG

To evaluate the robustness of the in-ear EEG recording system, we compare the power spectral density (PSD) between resting-states recorded with eyes closed and eyes open between both EEG systems. PSD was compared within five power bands: Delta (0.5–4 Hz), Theta (4–8 Hz), lower Alpha (8–10 Hz), upper Alpha (10–13 Hz), and Beta (13–30 Hz). To ensure a comprehensive comparison, first the results from the traditional Biosemi reference scheme will be presented, followed by the adapted Biosemi reference scheme and lastly the data recorded by the in-ear system will be presented. Resting-state analyses included all 19 participants for the BioSemi recordings. For the in-ear system, five datasets were excluded due to missing segments or excessive noise, resulting in *N* = 14.

### Biosemi traditional

**FIGURE 4:**
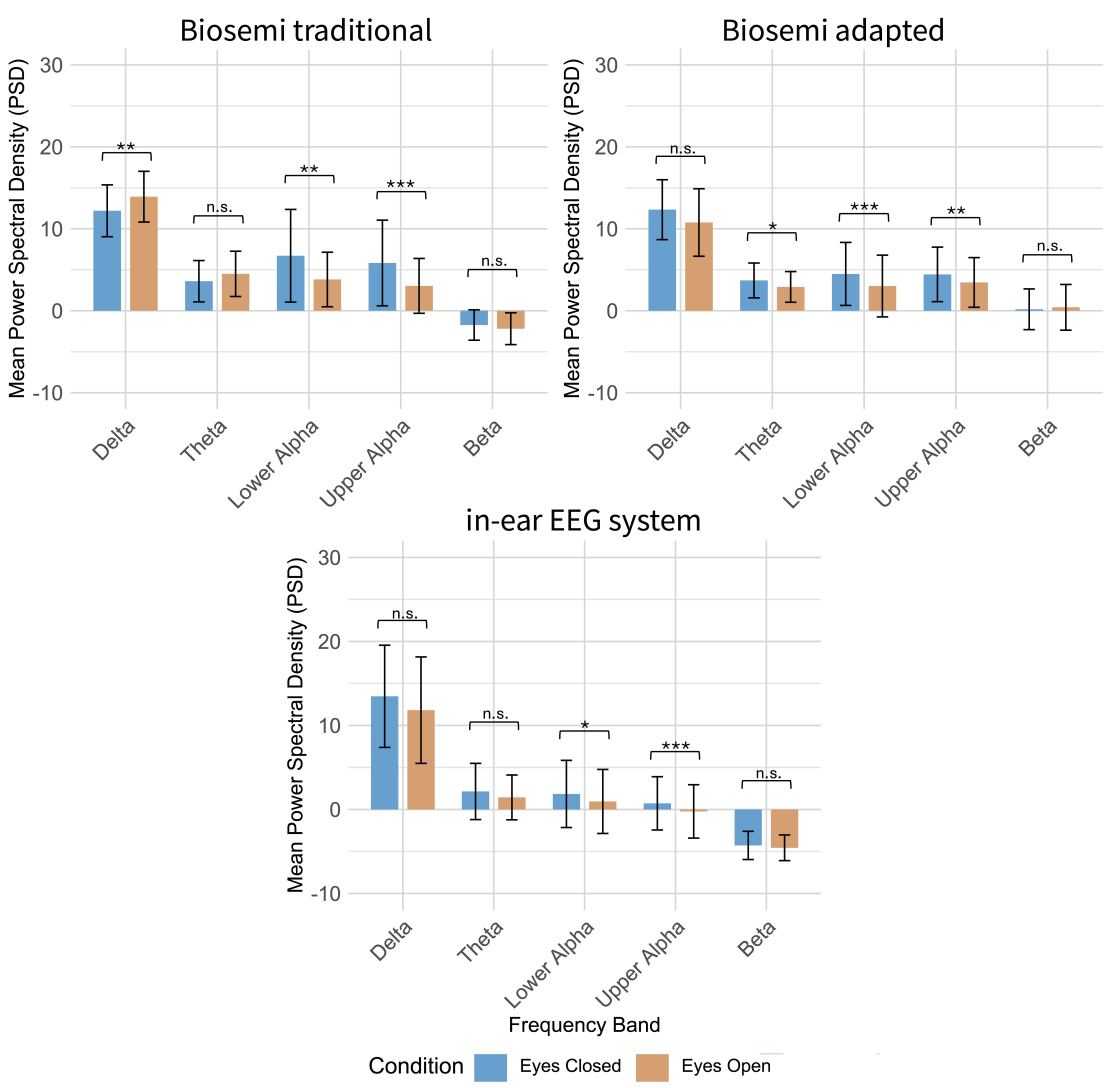
Differences in power spectral density between eyes closed and eyes open across frequency bands. Visualized are the results for both EEG systems, the upper panel reflects the data derived from the Biosemi System with both referencing schemes, the lower panel reflects the data derived from the in-ear device. n.s., not significant, *p < 0.05, **p < 0.01, ***p < 0.001.

Shapiro-Wilk tests indicated no significant deviation from normality across both conditions in all five frequency bands. As such, a paired sample *t*-test was appropriate to compare PSD across conditions. Paired sample *t*-tests revealed significant differences between eyes-closed and eyes-open conditions in the Delta [*t*(18) = − 3.65, *p* = 0.002, *d* = −1.72], lower Alpha [*t*(18) = 3.69, *p* = 0.002, *d* = 2.89], and Upper Alpha [*t*(18) = 4.30, *p* < 0.001, *d* = 2.79] bands. Delta band PSD was significantly lower in the eyes-closed condition (*M*_difference_ = −1.72, 95% CI [−2.71, −0.73]). Conversely, PSD in Lower Alpha (*M*_difference_ = 2.89, 95% CI [1.24, 4.54]) and Upper Alpha (*M*_difference_ = 2.79, 95% CI [1.43, 4.16]) bands were significantly increased during eyes-closed compared to eyes-open resting-state conditions. No significant differences were found for Theta band PSD [*t*(18) = −1.82, *p* = 0.086, *d* = −0.90] or Beta band PSD [*t*(18) = 1.39, *p* = 0.181, *d* = 0.45]. In summary, power spectral density significantly decreased during eyes closed in the delta range, but significantly increased in the alpha range, reflecting the expected dynamic of alpha power during resting-state. The results are summarized in Table 1 and visualized in Figure 4.

**TABLE 1:** Results from paired sample *t*-tests and Wilcoxon signed-rank test.

Frequency band	*T*	*df*	*p*	95% CI lower	95% CI upper	Δ *M*
Biosemi Traditional (paired-sample *t*-test)						
Delta	−3.65	18	0.002	−2.71	−0.73	−1.72
Theta	−1.82	18	0.086	−1.93	0.14	−0.89
Lower alpha	3.69	18	0.002	1.24	4.54	2.89
Upper alpha	4.29	18	< 0.001	1.43	4.16	2.79
Beta	1.39	18	0.182	−0.23	1.14	0.45
Biosemi adapted (Wilcoxon signed-rank test)						
Frequency band	V		*p*			
Delta	135		0.113			
Theta	151		0.023			
Lower alpha	176		< 0.001			
Upper alpha	165		0.003			
Beta	100		0.859			
In-ear EEG device (paired-sample t-test)						
**Frequency band**	***t***	***df***	***p***	**95% CI lower**	**95% CI upper**	**Δ *M***
Delta	1.36	13	0.198	−0.98	4.26	1.64
Theta	1.78	13	0.099	−0.15	1.56	0.70
Lower alpha	2.47	13	0.028	0.11	1.67	0.89
Upper alpha	4.98	13	< 0.001	0.54	1.37	0.96
Beta	2.02	13	0.064	−0.02	0.58	0.28

PSD between eyes closed and eyes open were compared.

### Biosemi adapted

As a next step, the same EEG data were analyzed, now with an adapted referencing scheme, using T7 as reference electrode and activity from T8 (reflecting a similar layout as the in-ear EEG device). Shapiro-Wilk tests indicated significant deviations from normality for some conditions, particularly PSD within the Delta band (eyes open: *W* = 0.87, *p* = 0.012), Theta (eyes closed: *W* = 0.89, *p* = 0.036), and Beta (eyes closed: *W* = 0.88, *p* = 0.020; eyes open: *W* = 0.86, *p* = 0.011) bands. Therefore, a non-parametric test was used to compare PSD across conditions.

Wilcoxon signed-rank tests revealed a significant difference between eyes-closed and eyes-open conditions in the Theta (*V* = 151, *z* = -3.18, *p* = 0.023), lower Alpha (*V* = 176, *z* = -2.82, *p* < 0.001), and upper Alpha (*V* = 165, *z* = -2.98, *p* = 0.003) bands. For all three frequency bands, PSD values were significantly increased in the eyes-closed condition compared to the eyes-open condition. No significant differences were found for Delta (*V* = 135, *z* = -3.42, *p* = 0.113) or Beta (*V* = 100, *z* = -3.92, *p* = 0.860). Taken together, the Biosemi system with adapted referencing scheme revealed a significant increase in theta and alpha band power during eyes-closed conditions compared to eyes open, consistent with the findings from the traditional referencing scheme. The results are summarized in Table 1 and visualized in Figure 4.

### In-ear EEG system

Lastly, the PSD derived from the in-ear system was analyzed and compared between eyes-closed and eyes-open conditions. Shapiro-Wilk test indicated no significant deviations from normality across all five frequency bands, with *p* > 0.05 for both conditions. Thus, parametric tests were deemed appropriate to compare PSD across conditions.

Paired sample *t*-tests revealed a significant difference in PSD between eyes-closed and eyes-open in the lower Alpha [*t*(13) = 2.47, *p* = 0.028, *d* = 0.89] and upper Alpha [*t*(13) = 4.97, *p* < 0.001, *d* = 0.96] bands. For both frequency bands, alpha power was higher during eyes-closed conditions (lower Alpha: *M*_difference_ = 0.89, 95% CI [0.11, 1.67]; Upper Alpha: *M*_difference_ = 0.96, 95% CI [0.54, 1.37]). No significant difference was observed for the Delta [*t*(13) = 1.36, *p* = 0.198], Theta [*t*(13) = 1.78, *p* = 0.099], or Beta [*t*(13) 2.02, *p* = 0.064] bands. In summary, the data derived from the in-ear device showed significant increase in alpha power during the eyes-closed condition. These results are highly consistent with those obtained from the Biosemi system, suggesting that the in-ear device can capture relevant neural dynamics during resting-state recordings. The results are summarized in Table 1 and visualized in Figure 4.

### Auditory evoked potentials

#### Data quality and epoch retention

Across participants, the BioSemi average-referenced Cz configuration retained a mean of 99.56% (SD = 0.66%) of epochs following automated artifact rejection. Baseline noise averaged 3.86 μV (SD = 0.71), with a median peak-to-peak amplitude of 18.99 μV (SD = 3.15). The adapted BioSemi T8–T7 configuration retained 98.38% (SD = 3.72%) of epochs. Baseline noise increased to 7.32 μV (SD = 1.30), and median peak-to-peak amplitude increased to 34.56 μV (SD = 7.02), reflecting reduced signal stability under constrained referencing. The in-ear configuration retained 93.98% (SD = 12.73%) of epochs. Baseline noise averaged 5.83 μV (SD = 2.47), with a median peak-to-peak amplitude of 28.32 μV (SD = 16.39), indicating intermediate signal variability relative to the two BioSemi configurations. ERP analyses included 17 participants for the BioSemi Cz configuration. For the in-ear system, three datasets were excluded, resulting in *N* = 16.

#### Component amplitudes

Using fixed time windows (N1: 125–175 ms; P2: 195–245 ms), the BioSemi Cz configuration showed a clear N1–P2 complex. The N1 component significantly deviated from baseline (*M* = -1.05 μV, SD = 1.25), *t*(16) = -3.49, *p* = 0.003. The P2 component showed a robust positive deviation (*M* = 2.81 μV, SD = 1.39), *t*(16) = 8.30, *p* < 0.001. In contrast, the adapted T8–T7 configuration did not yield significant deviations for either component (all *p* > 0.80), with mean amplitudes near zero (N1: -0.01 μV; P2: -0.05 μV). The in-ear configuration likewise did not show a significant N1 deviation (*M* = -0.05 μV, SD = 0.53), *t*(15) = -0.40, *p* = 0.694. The P2 component exhibited a non-significant trend in the negative direction (*M* = -0.50 μV, SD = 0.98), *t*(15) = -2.04, *p* = 0.060.

#### Component signal-to-noise ratio

**FIGURE 5:**
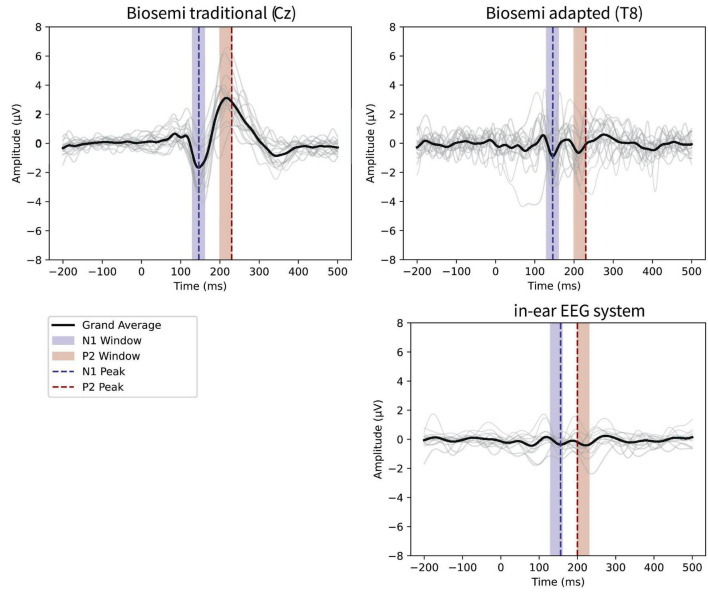
Grand average ERP across referencing schemes and devices. The upper panels depict the grand average auditory ERP computed from the data derived from the Biosemi system. The lower panel reflects the auditory ERP computed from the in-ear EEG system. The shaded blue and red areas reflect the time windows, and the dashed lines indicate the negative (blue) and positive (red) peak within each window.

Signal-to-noise ratio (SNR) analyses revealed a clear gradient across configurations. The BioSemi Cz configuration showed the highest SNR (N1: *M* = 0.32; P2: *M* = 0.74), followed by the T8–T7 configuration (N1: *M* = 0.10; P2: *M* = 0.10), and the in-ear configuration (N1: *M* = 0.07; P2: *M* = 0.12). These findings indicate progressively reduced detectability of time-locked responses as spatial sampling becomes more constrained. The results are summarized in Table 2 and visualized in Figure 5.

**TABLE 2:** Results from one-sample *t*-tests for N1 and P2 component against baseline across referencing schemes and devices.

Device	Component	*t*	Mean amplitude (μ V)	*p*
In-ear EEG	N1	-1.83	-0.23	0.094
In-ear EEG	P2	-1.69	-0.35	0.119
Biosemi (Cz)	N1	-4.56	-1.59	< 0.001
Biosemi (Cz)	P2	7.9	3.17	< 0.001
Biosemi (T8)	N1	-2.38	-0.66	0.029
Biosemi (T8)	P2	-1.11	-0.33	0.281

### Speech in noise

One final aim of the current study was to evaluate if an in-ear EEG device could reliably capture parietal alpha power modulations during adverse listening conditions, comparable to the response derived from a traditional scalp EEG System. To determine whether alpha power significantly deviated from baseline (ERSP = 0) in each noise condition and system, separate one-sample *t*-tests were conducted. Speech-in-noise analyses included 16 participants for the BioSemi recordings. For the in-ear system, three datasets were excluded, resulting in *N* = 16.

**FIGURE 6:**
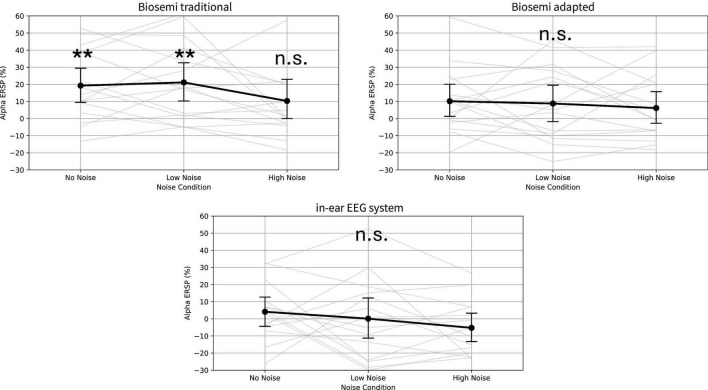
Grand average alpha ERSP deviations from baseline across conditions for each device and referencing scheme. Depicted are the grand average alpha ERSP changes (in %), the standard deviations, as well as the individual changes across both EEG systems and referencing schemes. n.s., not significant, *p < 0.05, **p < 0.01, ***p < 0.001.

For the traditionally referenced data derived from the Biosemi EEG system, a significant increase in alpha power was observed in the no noise condition [*t*(15) = 3.60, *p* = 0.003, *M* = 19.3] and in the low noise condition [*t*(15) = 3.52, *p* = 0.003, *M* = 21.2]. In contrast, alpha ERSP in the high noise condition did not significantly differ from baseline [*t*(15) = 1.75, *p* = 0.102, *M* = 10.3]. For the adapted referencing scheme within the Biosemi system, a similar but non-significant trend was observed. Alpha ERSP showed an increase in the no noise condition [*t*(15) = 2.03, *p* = 0.062, *M* = 10.2] almost reaching significance. However, neither the low noise nor the high noise condition was trending toward significant deviations (low noise: [*t*(15) = 1.54, *p* = 0.145, *M* = 8.8; high noise: *t*(15) = 1.23, *p* = 0.239, *M* = 6.16]. Lastly, no trends were observed within the data derived from the in-ear EEG system in any of the conditions (*p* > 0.24). The mean alpha ERSP and corresponding statistics are summarized in Table 3 and visualized in Figures 3, 6. In summary, a significant alpha power modulation in a parietal region was observed in traditionally referenced data derived from a Biosemi scalp EEG under less adverse listening situations. However, comparable significant deviations from baseline were not observed in the adapted referencing scheme or in-ear EEG configuration.

**TABLE 3:** Results from one-sample *t*-tests for N1 and P2 component against baseline across referencing schemes and devices.

Device	Noise level	*t*	Mean alpha ERSP (%)	*p*
Biosemi traditional	No noise	3.61	19.3	0.003
Biosemi traditional	Low noise	3.52	21.2	0.003
Biosemi traditional	High noise	1.75	10.3	0.102
Biosemi adapted	No noise	2.03	10.2	0.062
Biosemi adapted	Low noise	1.54	8.8	0.145
Biosemi adapted	High noise	1.23	6.2	0.239
In-ear EEG	No noise	0.92	4.1	0.374
In-ear EEG	Low noise	0.01	0.1	0.988
In-ear EEG	High noise	-1.21	-5.3	0.246

## Discussion

In this report, we provide a domain-specific evaluation of a fully in-ear, generic-fit EEG configuration relative to a conventional 32-channel scalp system (Biosemi ActivTwo) in healthy young adults (*N* = 19). Rather than testing equivalence between systems, our objective was to characterize the range of neural signals that can be robustly detected with this minimal hardware implementation and to identify boundary conditions where performance becomes constrained.

To this end, we examined three signal domains that differ in their physiological and analytical demands: (i) resting-state alpha modulation during eyes-open versus eyes-closed conditions, (ii) auditory event-related potentials (N1-P2 complex) elicited by pure tones, and (iii) alpha power modulation during a speech-in-noise listening task. Recordings were obtained simultaneously from the in-ear device and the scalp system, allowing direct benchmarking under identical physiological and environmental conditions. For the scalp EEG, two referencing schemes were applied: conventional average re-referencing and a T7-based re-referencing approach chosen to approximate the spatial configuration of the in-ear system.

### Alpha power modulation during resting-state

In the spectral domain, the fully in-ear configuration reliably captured canonical alpha modulation during resting state. Across both devices and all referencing schemes, alpha power (8–13 Hz) increased significantly during eyes-closed relative to eyes-open conditions, replicating the well-established state-dependent alpha enhancement observed in conventional scalp EEG (Barry et al., 2007; Hohaia et al., 2022; Su et al., 2021; Pfurtscheller et al., 1996). It has been suggested that the decreased activity within the visual cortex (due to closed eyes) allows for increased synchronization of neuronal activity, resulting in increased alpha power, which reflects an awake state without active processing of external stimuli (Pfurtscheller et al., 1996). However, there is also evidence supporting the functional role of alpha oscillation through inhibiting task irrelevant areas (Klimesch, 1999; Scheeringa et al., 2012; Foxe and Snyder, 2011). While the discussion of the underlying mechanisms that are responsible for the alpha power modulation is still ongoing, alpha power has been a highly prominent neural correlate of a broad range of cognitive functions (Pavlov and Kotchoubey, 2022; Schneider et al., 2022), sensory processing (Zhou et al., 2021), psychological (Roxburgh et al., 2023; Compton et al., 2019) and physiological components (Vanhollebeke et al., 2022).

Notably, absolute alpha power was generally lower in the in-ear recordings compared to the scalp system. Given the reduced spatial coverage, shorter inter-electrode distances, and fully intra-auricular referencing, a reduction in absolute amplitude is expected and does not necessarily imply reduced sensitivity to condition effects. Importantly, despite these amplitude differences, the relative modulation between eyes-open and eyes-closed conditions was preserved.

These findings suggest that minimal in-ear EEG is well-suited for paradigms relying on robust spectral contrasts, particularly within-subject designs aimed at monitoring neural state changes over time. Such configurations may therefore be advantageous in applications prioritizing wearability, rapid setup, and repeated measurements. At the same time, future hardware refinements that enhance electrode-skin coupling and spatial sampling could further increase signal amplitude and improve individual-level reliability.

### Auditory event-related potentials reflect a boundary condition of time-locked responses

In contrast to the spectral findings, the detectability of the auditory N1-P2 complex revealed clear boundary conditions for the minimal in-ear configuration. A robust N1-P2 response was reliably observed only in the scalp EEG using conventional average referencing. Neither the in-ear recording nor the scalp data using the T7-based reference yielded a comparably clear deviation from baseline within the expected component windows. Quantitative SNR estimates and baseline variability analyses indicated that residual noise remained high relative to the expected component amplitudes, limiting reliable component extraction despite automated artifact rejection and verified temporal alignment. Because the single-channel in-ear configuration did not permit ICA-based decomposition of ocular and other physiological artifacts, residual contamination may have further contributed to reduced signal-to-noise ratios. Importantly, this pattern is informative rather than unexpected. Early auditory ERPs are small-amplitude, phase-locked responses that require precise temporal stability, sufficient spatial sampling, and low baseline variability (Lightfoot, 2010). Unlike spectral power estimates, which integrate activity over time, time-domain ERPs depend critically on averaging large numbers of clean trials and preserving spatial sensitivity to underlying cortical generators. The N1-P2 complex is primarily generated in the auditory cortex and is typically maximal at fronto-central scalp sites (Tremblay et al., 2001). The fully in-ear configuration used here places all electrodes intra-auricularly, resulting in reduced spatial leverage relative to these generator regions and limited referencing flexibility. The fact that the adapted T7-based reference in the scalp system also reduced ERP detectability further underscores the importance of spatial configuration and referencing strategy. Thus, the limited N1-P2 observability in the in-ear system should not be interpreted as absence of neural responsiveness, but rather as a consequence of the interaction between component amplitude, spatial sampling geometry, and baseline noise characteristics.

These findings suggest that minimal in-ear EEG configurations may be less suited for capturing low-amplitude, early sensory ERPs under standard laboratory trial counts. Reliable estimation of such components may require substantially increased trial numbers, enhanced mechanical stabilization, improved electrode-skin coupling, and/or expanded spatial sampling. In contrast, later and higher-amplitude components, or frequency-domain steady-state responses, may be more resilient to these constraints, as they either benefit from stronger effect sizes or frequency-specific signal extraction methods (Kidmose et al., 2012; Goverdovsky et al., 2016).

Taken together, the ERP results delineate an important boundary: while spectral state changes are robustly measurable, small-amplitude transient responses impose stricter demands on spatial geometry and noise characteristics than the present minimal configuration can consistently satisfy.

### Speech in noise alpha modulation

The final aim of the current study was to assess whether an in-ear EEG system could detect parietal alpha modulation relative to baseline during speech-in-noise listening. A previous study by Ala et al. (2022) demonstrated a significant linear decrease in alpha power with increasing noise levels across both scalp EEG (using average and symmetrical referencing) and in-ear EEG. Our findings revealed that in the scalp EEG, using conventional average referencing, alpha power showed condition-dependent modulation consistent with prior work. In contrast, neither the adapted scalp reference nor the in-ear configuration yielded comparably robust effects.

Despite these differences, some aspects of our findings align with those reported by Ala et al. (2022). Specifically, our data suggest that alpha ERSP increases during easier listening conditions (no noise, low noise), while a significant modulation in the high noise condition was absent. These effects are consistent with previous studies demonstrating that alpha event-related synchronization may reflect auditory processing efficiency rather than solely listening effort (Seifi Ala et al., 2020; Wöstmann et al., 2017). However, the deviation in our results from the reported effects by Ala et al. (2022) are likely due to certain key methodological differences within the hardware set-up of the in-ear EEG system. Ala et al. (2022) used a customized in-ear EEG system with electrodes embedded in personalized earmolds, ensuring a stable and tight electrode fit for each participant. Their system included 12 active electrodes and incorporated active shielding which minimized external interference and motion artifacts. Additionally, their sampling rate was 1 kHz, enhancing the precision of spectral estimates. In contrast, our study utilized a fully in-ear EEG system containing only two active electrodes (right ear canal: signal electrode; left ear canal: reference electrode) and one passive ground electrode placed in the right concha. Unlike Ala et al., our system did not use active shielding, was not embedded in a customized earpiece and recorded data with a sampling rate of 250 Hz, which may have reduced the ability to detect fine-grained spectral modulations. Additionally, Ala et al. (2022) used a broader SNR range (-16, -8, -4, 4 dB), reflecting a wide spectrum of listening effort, while in our study a narrower SNR range (high noise: 3 dB, low noise: 8 dB, no noise) was used. While the broader range used by Ala et al. may have driven stronger modulations in alpha power, the lower overall task difficulty in our study may have limited the effect sizes.

Importantly, the absence of strong modulation in the in-ear data does not contradict the robust resting-state findings but rather suggests that spectral effects scale differently depending on effect size and artifact context. When alpha modulation is large and state-driven (e.g., eyes open vs. closed), the minimal configuration captures it reliably. When modulation is smaller, task-dependent, and embedded in a dynamic listening environment, detection becomes more sensitive to spatial sampling, referencing, and baseline variability.

These findings suggest that in-ear EEG may capture speech-related spectral dynamics under controlled conditions, but that robust estimation depends strongly on task design and noise characteristics. Increasing the range of signal-to-noise ratios, extending trial numbers, optimizing referencing strategies, and reducing movement-related artifacts may enhance detectability. More broadly, the results indicate that not all spectral paradigms translate equally well to minimal intra-auricular configurations, and that task selection should be guided by expected effect size and artifact profile.

## Limitations and design implications

The present findings should be interpreted within the constraints of the specific hardware configuration evaluated here: a fully intra-auricular, generic-fit system with two active electrodes and intra-ear referencing. This minimal geometry imposes inherent limitations on spatial sampling, inter-electrode distance, and referencing flexibility. As demonstrated in the ERP analyses, these factors disproportionately affect small-amplitude, time-locked responses that rely on high signal-to-noise ratios and spatial sensitivity to cortical generators.

Importantly, the reduced absolute signal amplitude observed in the in-ear recordings is consistent with physical and geometric constraints rather than indicative of absent neural responsiveness. Spectral state contrasts remained detectable despite these amplitude differences, underscoring that signal class and effect size interact with hardware configuration. Thus, the primary limitation identified here is not general feasibility, but domain-dependent sensitivity.

## Design recommendations

The boundary conditions delineated in this study yield several practical recommendations for future in-ear EEG research: *Match between paradigm and configuration specifics*. Robust spectral contrasts with large effect sizes (e.g., state-dependent alpha modulation) are well-suited to minimal intra-auricular systems. Small-amplitude transient ERPs require more favorable spatial sampling and lower baseline variability. *Optimize for SNR when targeting ERPs*. If early sensory components are primary endpoints, study designs should increase trial counts, minimize jaw and head movements, and prioritize electrode stability. Hardware refinements that enhance coupling consistency or expand spatial sampling may disproportionately benefit time-domain responses. *Report quantitative signal quality metrics*. Baseline variability, epoch retention, and component-level SNR should be systematically reported in in-ear EEG studies to distinguish hardware constraints from analytic variability and to facilitate cross-study comparability. *Develop configuration-aware paradigms*. Many existing EEG paradigms were optimized for multi-channel scalp systems. Future work should consider task designs that leverage the strength of intra-auricular configurations rather than directly transplanting paradigms designed for full-cap systems.

## Future directions

Future research should focus on configuration-specific optimization rather than general equivalence testing. On the hardware side, improvements in electrode-skin coupling stability, shielding, and multi-contact intra-auricular arrays may enhance spatial sensitivity without sacrificing wearability. On the methodological side, artifact modeling approaches tailored to the ear environment and paradigm designs calibrated to expected effect sizes may expand the range of reliably measurable signals.

## Conclusion

In summary, this study provides a domain-specific characterization of a fully intra-auricular, generic-fit EEG configuration benchmarked against a conventional 32-channel scalp system under identical recording conditions. Rather than testing equivalence, we delineated which classes of neural signals remain robust under a minimal hardware geometry and which impose stricter spatial and signal-to-noise requirements. Spectral state changes, particularly resting-state alpha modulation, were reliably captured by the in-ear configuration despite reduced absolute signal amplitude. In contrast, small-amplitude, time-locked auditory ERPs revealed clear boundary conditions, underscoring the importance of spatial sampling, referencing flexibility, and baseline stability for transient responses. Task-related alpha modulation during speech-in-noise fell between these extremes, highlighting that detectability scales with effect size and artifact environment.

Together, these findings demonstrate that reduced EEG systems do not scale uniformly across signal domains. By explicitly mapping strengths and constraints within a minimal intra-auricular configuration, the present work contributes to a principled framework for evaluating mobile EEG technologies. Such boundary mapping is essential for guiding study design, interpreting null findings, and informing future hardware and methodological development in wearable neurophysiology.

## Data Availability

The raw data supporting the conclusions of this article will be made available by the authors, without undue reservation.
